# Supplementary material for: Morning surge in blood pressure and sympathetic activity in Mongolians and Han Chinese: a multimodality investigation of hypertension and dyssomnia
Source: PeerJ. 2017 Sep 19;5:e3758. doi: 10.7717/peerj.3758 (PMC5609520; doi:10.7717/peerj.3758)
Supplement: Supplemental Information 2 [file peerj-05-3758-s002.docx]

**睡眠障碍对蒙族高血压患者交感兴奋性及晨峰血压的影响研究方案**

**一、研究背景及理论基础**

根据世界卫生组织估计，高血压每年导致全球710万人死亡，占全部死亡人口13%，亦是引致62%中风及49%心脏病发病的主因。据报告，到2025年，全球将有15.6亿人口患有高血压。据最新公布的《中国心血管病报告（2013）》显示，我国成人高血压患病率已达30%以上，高血压相关疾病死亡率排名第一。与该数字形成强烈反差的是高血压人群的知晓率、控制率很低，只有36%和28%。

目前高血压与睡眠相关临床研究主要聚焦在睡眠障碍与高血压的发病风险、血压昼夜节律的变化及高血压各种并发症发病及高血压治疗等方面。国外研究结表明睡眠障碍与高血压的发病风险、血压昼夜节律的变化及高血压各种并发症发病风险均有相关性，其中晨峰血压与心脑血管事件密切相关，睡眠质量可能是影响晨峰血压高低的主要因素。国内研究发现应用镇静睡眠药物改善患者睡眠质量，缓解患者焦虑情绪，有助于降压达标。

本研究目的采用匹兹堡睡眠质量指数( Pittsburgh Sleep Quality Index，PSQI)评估蒙汉族高血压病患者睡眠质量，采用24小时动态血压评估患者全天高血压情况，采用24小时尿儿茶酚胺检测评估交感神经全天的分泌情况，以探讨睡眠质量与交感神经兴奋性的关系，睡眠质量对蒙族高血压患者清晨血压的影响。

**二、研究目的**

1、分析蒙族高血压患者睡眠障碍的比例

2、评价蒙汉族高血压患者睡眠障碍与清晨血压的关系

3、蒙汉高血压患者睡眠障碍与交感活性的相关性分析

**三、研究方案设计**

本研究为横断面观察性研究，采用非随机调查研究的方案，预计纳入高血压组患者600余例。

**四、研究实施过程**

1、研究对象

从2016年11月开始选取包头医学院第二附属医院门诊就诊的蒙汉族高血压患者600余人作为入选对象，根据病情描述、化验检查及判定标准由专科医生确诊为原发性高血压患者。根据入选及排除标准进入本研究。

入选标准 **:** （1）年龄≥18岁蒙汉族高血压患者；(2)非同日测量3次或3次以上血压收缩压≥140mmHg或舒张压≥90mmHg并血压<180/110mmHg；(3)1周内未服用过任何降压药物。

**(1) The Mongolian and Han hypertensive patients with age ≥18 years old;**

**(2) The patients with the systolic blood pressure ≥140mmHg or diastolic blood pressure ≥ 90mmHg and blood pressure <180/110mmHg were measured ≥3 times in different day.**

**(3) Have not take any antihypertensive drugs within the last week.**

排除标准：（1）继发性高血压患者 （2）并发脑卒中、冠心病、糖尿病、严重的心律失常、心衰、瓣膜病（3）肝肾功能不全（4）精神异常（5）肿瘤患者 （6）呼吸系统疾患（7）术后3个月之内（8）甲亢（9）结缔组织病(10)妊娠、脯乳期(11) 感染（12）不能自主完成调查问卷(13）不愿意参加该临床研究的患者。

2研究方法

1. 所有受试者完成匹兹堡睡眠质量指数评分：每位受试者根据自己近1个月睡眠情况填写匹兹堡睡眠质量指数评分（PSQI）表,PSQI>7分为睡眠功能障碍。睡眠功能障碍者进入下列检查。
2. 人口基本资料调查表，内容包括：姓名，性别，年龄，身高，体重，居住地址及文化程度等。

(3）生化指标检测：抽取受试者晨起空腹肘静脉血，化验：血常规、血糖、血脂、钾钠氯、肝功、肾功等。

(4)24h ABP 监测：采用无创性动态血压监测仪 (日本 欧姆龙HEM-9000AI)进行 24 h 动态血压监测。日间 (6: 00-22: 00) 每30 min 测量1 次，夜间 (22: 00-次日6: 00) 每小时测量 1 次。 监测当天记录受试者日常生活及起居情况, 监测次数不少于额定次数80%的血压数据才予以分析。

（5）尿儿茶酚胺检测：患者标本的收集：早7点至次日7点的尿液收集到一个清洁的大容器内，第一个7点的尿弃去，第二个7点的尿留下，在留尿前放入相应的防腐剂，24h尿液留好后，搅匀后留取标本10ml,并量总量写于化验单上送化验室检测。

**五、研究步骤：**

1 筛选入选对象→匹兹堡睡眠质量指数评分>7→进行生化指标检查→24h ABP 监测→尿儿茶酚胺测定。

2研究对象分组：根据民族分组；根据匹兹堡睡眠质量指数评分分组。

3分析蒙汉族不同睡眠质量指数评分、入睡时间、睡眠时间、睡眠质量时，夜间平均压、日间平均压、晨峰血压及尿儿茶酚氨间的关系；分析蒙汉族勺型高血压和非勺型高血压组睡眠质量指数、尿儿茶酚氨的关系，分析蒙汉族不同年龄段、不同性别间睡眠质量指数、尿儿茶酚氨的关系。

**六、研究的风险/收益**

本研究为观察性研究，不会带来不适和额外风险，匹兹堡睡眠质量指数评分和24小时动态血压检测作为一种无创、快速的睡眠、血压评价方法，可能会很好的评价清晨高血压情况，使更多的高血压患者收益。

**七、受试者的招募和保护措施**

受试者为门诊高血压患者，采用自愿参与的原则，在试验流程进行的过程中，尽可能保证患者安全及隐私。

**八、知情同意过程**

由课题组研究人员负责患者的招募，并负责患者的知情同意过程，如患者不具备知情同意的能力，则排除此患者；在知情同意的过程中，严格遵守自愿的原则，不胁迫或引诱患者参加试验。在参与研究过程中，尽量保证研究参与者及时得到信息（包括他们的权利、安全和健康）。

**九、费用**

匹兹堡睡眠质量指数评分、24h ABP 监测和尿儿茶酚胺检测完全免费，其他相关临床检查费用按照主城区普通医保的报销额度和比例进行报销。医生将尽全力预防和治疗由于本研究可能带来的伤害。如果在临床试验中出现不良事件，医学专家委员会将会鉴定其是否与本试验相关操作有关。申办者将按照我国临床试验的相关规定对与试验相关的损害提供治疗的费用及相应的经济补偿，具体补偿方案将根据具体情况决定。

**十、数据的获得和质控**

匹兹堡睡眠质量指数评分受试者填写完后可直接获得，数据记录不完整的退回受试者填写完整或电话询问完成表格，无法填写完整的评分表排除本研究；24h ABP 监测可通过日本欧姆龙公司软件直接分析得到，监测次数少于额定次数80%的血压数据排除试验外；尿儿茶酚胺检测，化验标本未按要求留取尿液的排除试验之外。

**十一、有关受试者的信息和保密**

患者的医疗记录将完整地保存医院。研究者、伦理委员会和医疗监督管理部门将被允许查阅换的医疗记录。任何有关本项研究结果的公开报告将不会披露患者的个人身份。工作人员将在法律允许的范围内，尽一切努力保护患者个人医疗资料的隐私。

**十二、成果分享**

研究结束后，成果将以论文的形式公开发表。
