# Supplementary material for: Morning surge in blood pressure and sympathetic activity in Mongolians and Han Chinese: a multimodality investigation of hypertension and dyssomnia
Source: PeerJ. 2017 Sep 19;5:e3758. doi: 10.7717/peerj.3758 (PMC5609520; doi:10.7717/peerj.3758)
Supplement: Supplemental Information 4 [file peerj-05-3758-s004.doc]

**
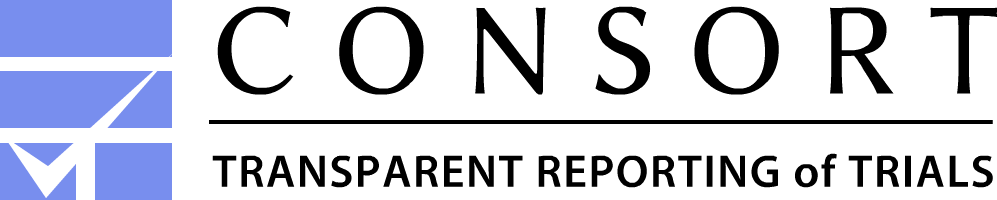
**

**CONSORT 2010 Flow Diagram**

**Allocation**

**Analysis**

**Follow-Up**

**Enrollment**

Assessed for eligibility (n=692 )

Excluded (n=436 )

♦  Not meeting inclusion criteria (n=411 )

♦  Other reasons (n=25 )

Analysed (n=87 )
The detailed procedure is shown in Figure 1

Lost to follow-up (give reasons) (n=23 )

Discontinued intervention (give reasons) (n=87 )

Allocated to Mongolian group (n=110 )

Lost to follow-up (give reasons) (n=31 )

Discontinued intervention (give reasons) (n=115 )

Allocated to Han Chinese (n=146 )

Analysed (n=115 )
The detailed procedure is shown in Figure 1

Selected subjects(n=256 )
